# Protocol for the detection of large dense-core vesicle exocytosis using an automated image-processing algorithm

**DOI:** 10.1016/j.xpro.2025.104264

**Published:** 2025-12-10

**Authors:** Aishwarya Makam, Vishnu Ramadas, Anly Tollan, Ishan Bhattacharyya, Abhimanyu Dubey, Nikhil R. Gandasi

**Affiliations:** 1 Cell Metabolism Lab (GA-08), Department of Developmental Biology and Genetics (DBG), Indian Institute of Science (IISc), Bengaluru 560012, India; 2 Department of Chemical Engineering, Indian Institute of Science (IISc), Bengaluru 560012, India; 3 Department of Medical Cell Biology, Uppsala University, BMC 571, 75123 Uppsala, Sweden

**Keywords:** Cell biology, Metabolism, Microscopy

## Abstract

Investigating exocytosis in human pancreatic islet cells is challenging due to small vesicle size and variable imaging parameters. Here, we present a protocol to detect and analyze exocytosis with an image-processing algorithm using Lagrangian particle tracking. We describe steps for sample preparation, total internal reflection fluorescence (TIRF) microscopy imaging, and computational analysis. The algorithm is validated by mathematical models, TetraSpeck beads, and cell images. Its applicability to other cellular processes and its handling of large sets of data make it useful for high-throughput microscopy research.

For complete details on the use and execution of this protocol, please refer to Makam et al.^1^

## Highlights

• Instructions for isolation, culture, and transfection of human pancreatic islet cells
• Steps for preparation of beads and artificial images for algorithm validation
• Guidance on TIRF imaging of exocytosis events in human pancreatic islet cells
• Procedure for detecting and analyzing vesicle dynamics using Lagrangian tracking

## Abstract (editor highlights)

Publisher’s note: Undertaking any experimental protocol requires adherence to local institutional guidelines for laboratory safety and ethics.

## Graphical abstract

**Figure:**
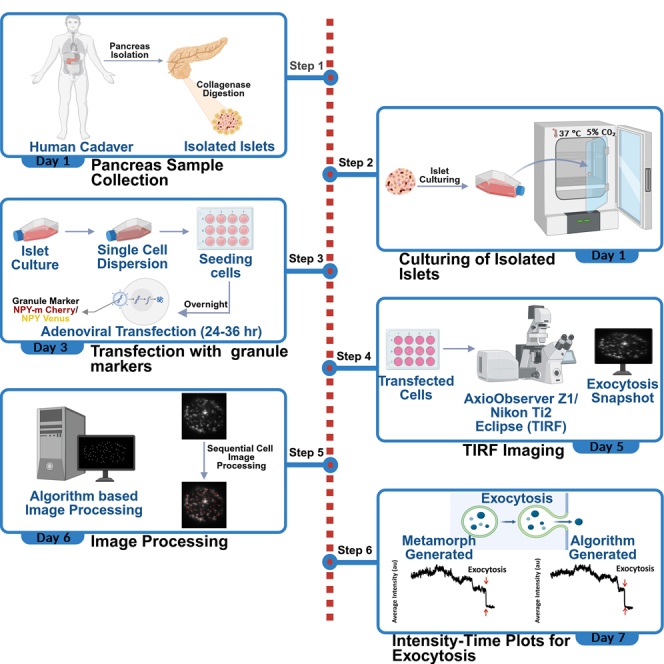

## Before you begin

The biggest challenge of this task is to come up with an effective, versatile image-processing tool for analyzing exocytosis, a vital cellular process that deals with the release of vesicular contents.^2^^,^^3^ As vesicles are small in size and there are a lot of variances in imaging parameters, current tools are generally lacking in terms of precision and speed.^1^^,^^4^ We set out to design an efficient Lagrangian particle tracking algorithm with high sensitivity for accurately locating punctate structures. During exocytosis, the punctate structures disappear with numerous changes in intensity; therefore, effective processing of large sets of data is essential.^5^ Validation against artificial images, fluorescent beads, and live cell images enables us to provide an enhanced method better suited to existing methods and achieve rapid, high-throughput analyses of exocytosis as well as other cell-based dynamics.^1^^,^^4^^,^^6^

Ensure all ethical clearances are in place for handling human pancreatic islets and verify that institutional and regional regulations are met.^6^ Prepare all reagents needed, such as CMRL 1066 culture medium, imaging buffer, and cell dissociation buffer, in a sterile form, and store them in proper conditions. Calibrate TIRF microscope, verify laser alignment.^7^^,^^8^ Prepare adenovirus stocks for granule markers (e.g., NPY-Venus or NPY-mCherry) and prepare Lipofectamine 2000 for transfection.^9^^,^^10^ Install and familiarise yourself with the Lagrangian particle tracking algorithm to verify compatibility with your imaging setup.^5^^,^^9^^,^^11^^,^^12^^,^^13^ Follow biosafety level 2 (BSL-2) protocols when handling human tissue and viral vectors and dispose of biohazardous waste according to institutional policy.^14^ These preparations are crucial to the protocol’s smooth operation and prevention of delays.

### Institutional permissions

All experimental protocols adhere to the policies of the Nordic Network for Clinical Islet Transplantation, ADI Isletcore at the University of Alberta and the Indian Institute of Science. Before beginning the experiments and following the procedures outlined in this protocol, it is critical to ensure that the required permissions from the relevant institutions, including patient/guardian consent, have been acquired.

### Innovation

In the study, we describe automated analysis of imaging-based detection of exocytosis. Although many image analysis-based plugins existed for detection of membrane trafficking in general in live cells, very few work well for exocytosis specifically. Exocytosis, when visualized under a microscope, has a unique increase in fluorescence coupled with a complete abrupt loss of signal. The first step to detect such events is to detect labelled granules with high fidelity, so that we can follow a fraction of them that later undergo exocytosis. This protocol is unique since it follows Lagrangian particle tracking algorithm to detect granules with high fidelity across various time points, so changes during exocytosis can be detected. Another important advancement is the ability of the algorithm to detect exocytosis events from primary tissue, for example, in this case, the islets of Langerhans. Such a protocol would save immense time for researchers working on exocytosis using imaging-based methods and ensure a standard way across different such studies to obtain comparable data.

## Key resources table

**Table:** 

REAGENT or RESOURCE	SOURCE	IDENTIFIER
**Biological samples**		

Human islets	Nordic Network for Clinical Islet Transplantation and ADI Isletcore at the University of Alberta	https://nordicislets.medscinet.com/en.aspx,https://www.bcell.org/adi-isletcore.html

**Cell culture reagents and media**		

CMRL 1066 culture medium	Thermo Fisher Scientific	21540026
5.5 mM glucose	Thermo Fisher Scientific	A2494001
10% fetal bovine serum	Thermo Fisher Scientific	A5256701
2 mM L-glutamine	Thermo Fisher Scientific	25030081
Penicillin-Streptomycin (1,000 U/mL)	Thermo Fisher Scientific	15140122
Ca^2+^-free cell dissociation buffer	Thermo Fisher Scientific	13151014
0.5% trypsin EDTA (10×)	Thermo Fisher Scientific	15400–054
Lipofectamine 2000	Thermo Fisher Scientific	11668027
100 nm TetraSpeck beads	Thermo Fisher Scientific	T7279
Poly-L-Lysine	Sigma-Aldrich	P8920-100ML

**Plasmid**		

NPY-mCherry, NPY-GFP	Addgene	74629, 67156

**Buffers**		

NaCl	Sisco Research Laboratories	41721(CAS-7647-14-5)
KCl	Sisco Research Laboratories	84984(CAS-7447-40-7)
MgCl_2_	Sisco Research Laboratories	13546(CAS-7791-18-6)
CaCl_2_	Sisco Research Laboratories	84336(CAS-10043-52-4)
NaOH	Sisco Research Laboratories	68151(CAS-1310-73-2
HEPES	Thermo Fisher Scientific	15630080
D-glucose	Sisco Research Laboratories	42738 (CAS-50-99-7)
PBS pH 7.4(1×)	Thermo Fisher Scientific	10010–023

**Software and algorithms**		

Fiji (ImageJ) with Find Maxima plugin	NIH	https://imagej.net/software/fiji/downloads
Metamorph	Molecular Devices	https://www.moleculardevices.com/products/cellular-imaging-systems/high-content-analysis/metamorph-microscopy
MATLAB	MathWorks	Version: R2025a
GraphPad Prism 8.0	GraphPad Software, Inc.	https://www.graphpad.com/

**Others**		

22 mm Coverslip	Bluestar	BSS310
6-well Tissue Culture (TC)-treated clear flat-bottom polystyrene plate with round clear wells, sterile	Corning	CLS3516
TIRF microscope	Nikon Instruments	Nikon Ti2 E
Objective lens	Nikon Instruments	
Camera	Caim Research Ltd.	Photometrics evolve 512 delta
Laser	TOPTICA Photonics AG, Graefelfing, Germany	Product ID: iChrome MLE-L-CD_30497 Version: .05

## Materials and equipment

**Table:** CMRL 1066 Medium

Reagent	Final concentration	Amount
CMRL 1066 (base)	N/A	≈88 mL
Glucose	5.5 mM	0.55 mL
Fetal calf serum	10%	10 mL
L-Glutamine	2 mM	1 mL
Streptomycin	100 U/mL	0.5 mL
Penicillin	100 U/mL	0.5 mL
Total	N/A	100 mL

**Table:** 1× Trypsin (0.05% solution)

Reagent	Final concentration	Amount
0.5% trypsin	10×	1 mL
PBS	1×	9 mL
Total	N/A	10 mL

### Microscopy and image acquisition

Total Internal Reflection Fluorescence (TIRF) microscopy was used to visualize exocytotic events occurring at the plasma membrane. Live-cell imaging was performed using an AxioObserver Z1/Nikon Ti2 Eclipse TIRF microscope equipped with a 100× objective (NA 1.49) and an EMCCD camera (Photometrics evolve 512 delta). Images were captured at an acquisition rate of 20 frames per second with an exposure time of 50 ms. Excitation was performed using a 488 nm laser for GFP-tagged vesicles.

## Step-by-step method details

### Human pancreatic islet cell preparation

**Timing: 3 weeks**

This section helps in the preparation of human islets post-isolation for further imaging-related experiments.

1. Isolation and culture of human pancreatic islets
  a. Obtain human pancreatic islets from cadaveric donors with ethical approval and written donor consent.
  b. Culture the islets in CMRL 1066 medium supplemented with 5.5 mM glucose, 10% fetal calf serum (FCS), 2 mM L-glutamine, 100 U/mL penicillin, and 100 U/mL streptomycin.
  c. Cultures are maintained at 37°C in a humidified incubator with 5% CO_2_ for approximately two weeks.

**CRITICAL:** It is essential to maintain the islets in a sterile condition during this recovery period. Alternative reagents like primosin can be used to avoid contamination.

2. Dispersion of Islets into Single Cell
  a. Islets are washed twice with Ca^2+^-free cell dissociation buffer.
  b. Treat the islets using 1× trypsin (0.05%) and incubate at 37°C for 5–10 min with gentle agitation.
  c. Add serum-containing medium to stop the reaction.
  d. Detach the cells and transfer them into a corning conical centrifuge tube.
  e. centrifuge the cells at 300 × g to pellet the cells.
  f. Resuspend the pellet with fresh serum-containing medium.

**CRITICAL:** It is important to maintain the duration of trypsinization. Over trypsinisation may lead to degradation of the cells and prove detrimental to islet health.

3. Plating and transfection
  a. Rinse 22 mm glass coverslips with 70% ethanol and leave to air dry.
  b. Place the dried coverslips into a six-well plate.
  c. Prepare a working solution of Poly-L-Lysine (0.01% w/v in PBS) ≈ 500 μL per well in a 6-well plate.
  d. Add Poly-L-lysine solution to each well to completely cover each coverslip and incubate for 20 min at 25°C.
  e. Rinse the coverslips 2–3 times with PBS and leave to air-dry completely in a sterile laminar flow hood.
  f. Seed cells onto the Poly-L-Lysine coated coverslips at a density of 50,000–100,000 cells per coverslip.
  g. Incubate the cells 12-16 h in a 37°C, 5% CO_2_ incubator, to allow cells to attach onto the coverslips.
  h. Transfect the cells by adding 100 μl of OptiMEM containing 0.5 μg plasmid DNA (granule markers such as NPY-GFP or NPY-mCherry) and 0.5 μl of lipofectamine 2000. After 5-6 h of incubation, stop the transfection by replacing the medium with a complete serum-containing medium.
  i. Optional: Transfect cells with cDNA encoding eGFP-tagged proteins using Lipofectamine 2000.
  j. Image cells 24–36 h after transfection to allow recovery.

**CRITICAL:** It is important to use OptiMEM during transfection to avoid stressing the cells. Alternatively, low serum-containing media can be used. Lipofectamine can be replaced by any other liposome-based transfection reagent which provides good transfection efficiency.

### Preparation of beads and artificial images

**Timing: 4 h**

This section helps in the preparation of beads and artificial images important for the validation of the algorithm.

**Figure 1:**
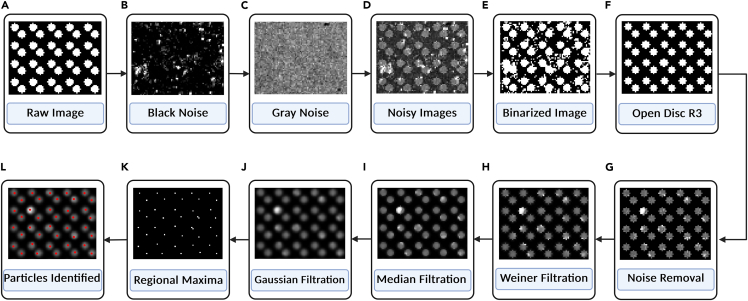
Steps involved in the generation of artificial images (A–G) Creation of circular objects and addition of noise. (H–L) Filtration and particle identification using find maxima.

4. Immobilization of Fluorescent Beads
  a. Dilute 100 nm TetraSpeck beads at a 1:100 ratio in 1×PBS.
  b. Add 10 μL of the diluted beads onto a 22-mm coverslip.
  c. Allow the beads to settle for 10 min at 25°C.
  d. Gently wash 2-3 times with 1×PBS to remove unbound beads.
  e. TIRF microscopy can be used to image the beads.
5. Generation of Artificial Images
  a. Create circular objects using the equation
    i. (x±x1)^2^+(y+y1)^2^≤r^2^ where (x,y) – circle’s centre co-ordinates and r denotes the radius.
  b. Change the radius to create different vesicle areas.
  c. To represent differing vesicle sizes (distinguished, tangent, intersecting) apply differing radius r.
    Make several noisy versions (e.g., Gaussian, Poisson) and normalize all images to [0, 1].
  d. Final Image Composition: Final= w_1_.Noise_1_ + w_2_.Noise_2_ + (1-(w_1+_w_2_)).Raw
  e. Change several parameters (e.g., radius, weight of noise) to mimic TIRF images (Figure 1).

### Induction of exocytosis

**Timing: 10 min**

This section details the induction of exocytosis in cultured islets.

6. For induction of exocytosis, prepare extracellular imaging buffer containing (in mM): 138 NaCl, 5.6 KCl, 1.2 MgCl_2_, 2.6 CaCl_2_, 10 D-glucose, and 5 HEPES (pH 7.4 with NaOH). Supplement with 200 μM diazoxide and 2 μM forskolin.^15^^,^^16^^,^^17^
7. Then apply a 75 mM KCl solution (prepared by equimolarly replacing NaCl in the buffer) locally using a pressurized glass electrode to evoke exocytosis.^15^^,^^18^
8. Maintain cells under constant perfusion with the exocytosis buffer at 32 °C during imaging.

### Microscopy

**Timing: 3–4 h**

This section explains the microscopic examination of exocytosis events.

9. TIRF Microscopy Setup
  a. Set up the TIRF microscope with a 100×/1.45 objective.
  b. Samples will be excited with 491/488 nm lasers.
  c. Emission light is captured with an EMCCD camera (Photometrics evolve 512 delta).
  d. Keep the scaling to 160/130 nm per pixel.

### Image processing and analysis—Granule quantification and analysis pipeline using algorithm

**Timing: 1 day**

This section enables the analysis of the images using the by-eye and find maxima-based detection methods.

10. By eye analysis
  a. Image analysis: Count the particles manually using Metamorph.
  b. Quantification: Record the total number of particles per image.
  c. Data processing: Calculate the averaged values based on individual images captured.
  d. Statistical analysis: Calculate the standard error of mean (SEM) for each condition. `
11. Find Maxima
  a. Calculate the granule density using the built-in plugin ’Find Maxima’ in ImageJ (Figure 2)

**Figure 2:**
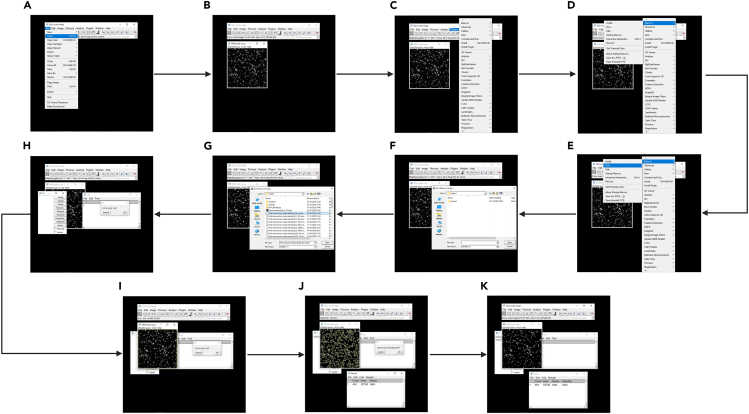
Steps involved in the find maxima-based detection (A–C) Loading the image onto Fiji(ImageJ). (D–G) Selection of the macros and the find maxima plugin to be used. (H–K) Circling of the cell (ROI) and particle detection.
  b. Quantification: Record the total number of particles.
  c. Data processing: Average out the values obtained from individual images.
  d. Statistical analysis: Calculate the standard error of the mean (SEM) for each condition.
12. Algorithm
  a. Load acquired images into the algorithm.
  b. Detect punctate structures (vesicles) and quantify intensity changes.
  c. Compare results with ImageJ - Find Maxima and manual detection for validation.

### Image-processing algorithm

**Timing: 2 h**

The section outlines the workflow of the image-processing algorithm for detecting exocytosis events (Figure S1).

13. Image Pre-processing
  a. Apply a Wiener filter to the raw images which helps to enhance the signal-to-noise ratio (SNR) while preserving edges.
    i. This step improves image clarity and prepares the data for subsequent processing.

**Figure 3:**
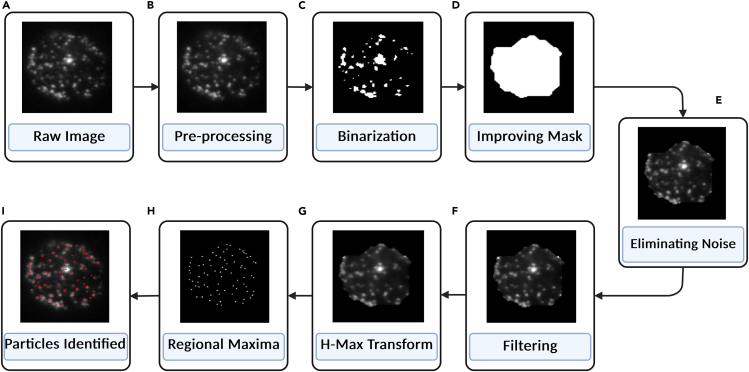
Sequential steps involved in the workflow of the image processing algorithm (A and B) Image pre-processing. (C and D) Binarization and processing of the mask. (E and F) Noise removal and filtration. (G–I) Regional Maixma and particle detection.

Refer to Figure 3B for a visual representation of the pre-processed image.

14. Binarization
  a. Convert the pre-processed image into a binary format - Otsu’s Algorithm evaluates the threshold using zeroth and first-order cumulative moments.
  b. Address spatial intensity variations by dividing the image into smaller sub-images (32 × 32 pixels) and applying Otsu’s Algorithm to each sub-image.

***Note:*** If low-intensity pixels are not detected, reduce the sub-image size to 16 × 16 pixels or manually adjust the threshold for specific sub-images.

Refer to Figure 3C for the binarized image.

15. Pre-Processing of Mask
  a. Refine the binary mask to isolate specific cell clusters using morphological closing operations (dilation followed by erosion) - Use a disc structuring element with a radius of 100 pixels to bridge larger gaps.
  b. Dilate with a disc of radius 5 ± 3 pixels.
  c. Apply an opening operation with a disc of radius 3 ± 1 pixels.

Refer to Figure 3D for the refined mask.

16. Noise Removal and Filtering
  a. Overlay the refined mask onto the original raw image to isolate cell clusters and set background pixels to zero.

Refer to Figure 3E for the masked image.

b. Scale the intensity values of the masked image between 0 and 1.
c. Visualize the image matrix as a surface plot, with intensity values representing height at each (x, y) position.
  i. Use the Regional Maxima finding algorithm to identify vesicles as local peaks.
d. Apply a series of filters to reduce noise and enhance vesicle detection:
  i. Wiener filter
  ii. Sharpening filter
  iii. Gaussian filter

Refer to Figure 3F for the filtered image.

e. If over-segmentation occurs, apply the H-Max Transform to suppress smaller peaks and reapply the Regional Maxima algorithm.

Refer to Figure 3G for the image after H-Max Transform.

17. Detection of Dynamic Events
  a. Identify particle positions across all images in a stack.
  b. Calculate intensity values by averaging the intensity at the particle position and its four neighboring points.
  c. Use the tracker to construct particle trajectories over time, revealing vesicle movement and intensity changes.
    ***Note:*** If no significant motion is detected, use the first frame to determine vesicle positions and calculate intensities for subsequent frames.
  i. Plot intensity values over time and create surface plots for visualization.

Refer to Figure 3I for the final vesicle positions.

18. Statistics.
  a. Present data as mean ± standard error of the mean (s.e.m.).
  b. Assess statistical significance using One-way ANOVA.

## Expected outcomes

**Figure 4:**
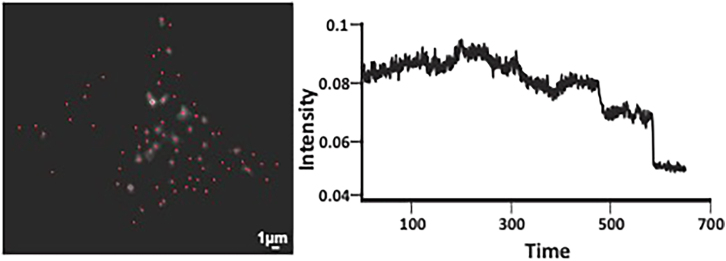
Detection of exocytosis using the algorithm (left) The identification of the vesicles in all the frames of the time-lapse movie. (right) Detection of exocytosis events based on changes in intensities in the marked coordinates used to identify vesicles.

Images and movies of transduced human islets using a high-resolution TIRF microscope would enable the algorithm to identify vesicles (Figure 4, left). Step 6e would enable the detection of exocytosis, as shown in Figure 4, right.

**Figure 5:**
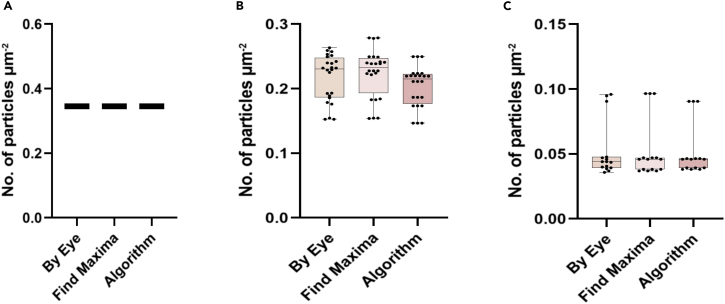
Accuracy plots showing the ability of the algorithm to detect particles (A–C) The Algorithm was able to detect efficiently the number of particles in A. the artificial images B. the beads images C. the real cell images in comparison with manual, by-eye detection and find maxima-based detection using Fiji/ImageJ (P-value>0.1).

**Figure 6:**
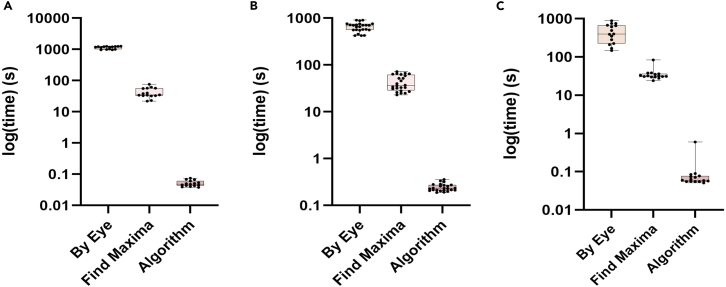
Time plots showing the efficiency of the algorithm to complete tasks accuratly and quickly (A–C) Plots showing the average time taken for the detection of the particles using the three techniques for A. artificial images B. beads images C. real cell images. In all the cases the algorithm was shown to take the significantly less amount of time when compared to the other two techniques (P-value<0.001).

The ability of the algorithm to detect particles in images of cells with low signal-to-noise ratio was built gradually by training it to detect particles in artificially ordered images, followed by images of Tetraspeck beads of a known size. The plots in Figure 5 show the accuracy of the algorithm to detect particles in the above-mentioned scenarios when compared to manual by eye-based and find maxima-based detection. The average time the algorithm takes to detect the particles will be significantly less than the other two methods, as shown in Figure 6.

## Limitations

The algorithm’s detection is not suitable for images of Hematoxylin and eosin-stained sections of tissues or images from immunohistochemistry sections. Intracellular structures with a size range like the vesicles used here can be detected very efficiently, whereas processing parameters might have to be altered to enable the detection of particles with varying sizes. The exocytosis detection for relatively stationary particles is very detected with utmost precision here. In contrast, tracking of particles beyond a certain limit would not be possible, since this algorithm does not use the usual tracking-based method of exocytosis detection. While this has several uses, this limits the ability of the algorithm to detect extremely active movement of vesicles.

The fluorescent probes used for detection of exocytosis can show difference in fluorescence intensity patterns. Either the parameters of this algorithm have to changed based on probe, or there are many algorithms available based on different fluorescent probe.^4^^,^^19^^,^^20^^,^^21^^,^^22^^,^^23^^,^^24^^,^^25^^,^^26^^,^^27^^,^^28^

## Troubleshooting

### Problem 1

Improper islet dispersion (step 1b).

### Potential solution

Islet dispersal is a critical step, and over- or under-dispersal will affect the viability of the cells for further procedures. This problem can occur due to improper digestion of the tissue. Hence, the collagenase concentration and treatment duration need to be determined based on the amount of tissue. Another stage at which this problem can occur is during trypsin digestion of the isolated islets. The amount of trypsin to be used and the incubation time need to be calculated based on the amount of tissue and the permeabilization extent of the tissue.

### Problem 2

Lack of fluorescence-positive cells (step 1c).

### Potential solution

While using the adenoviral-mediated method of labelling proteins, the MOI can be tweaked in order to achieve the ideal efficiency of positive cells. The ratio between the transfection reagent and the plasmid of interest needs to be standardized while using the transfection-based strategy.

### Problem 3

Inability to detect the appropriate number of intracellular structures (vesicles) (step 5a).

### Potential solution

The particle in question might be of a different diameter than what this algorithm has been made for. To be able to detect particles in datasets different from ours, the kernel size can be adjusted in the code. This would efficiently help in identifying particles of varying size range.

## Resource availability

### Lead contact

Further information and requests for resources and reagents should be directed to and will be fulfilled by the lead contact, Nikhil R. Gandasi (grnikhil@iisc.ac.in, nikhil.gandasi@mcb.uu.se).

### Technical contact

Technical questions on executing this protocol should be directed to and will be answered by the technical contact, Aishwarya Makam (aishwaryama@iisc.ac.in).

### Materials availability

This study did not generate new materials.

### Data and code availability

• The MATLAB code for the algorithm data has been deposited at Github: https://github.com/dabhimanyu/bio_math_model_images.git.
• Any additional information for reanalyzing this work is available from the lead contact upon request.
• The version of record of the MATLAB pipeline used in this study is archived on Zenodo: https://doi.org/10.5281/zenodo.17342495 and corresponds to the GitHub release (v.1.0 doi) (Github: https://github.com/dabhimanyu/bio_math_model_images/releases/tag/v1.0-doi).

## Acknowledgments

This research was funded by the 10.13039/100007780Indian Institute of Science – seed grants, 10.13039/501100001407Department of Biotechnology (DBT)-Ramalingaswami fellowship, 10.13039/501100001411Indian Council of Medical Research (ICMR) – Grants in Aid Scheme, 10.13039/501100001409Department of Science and Technology (DST)-10.13039/501100001843Science and Engineering Research Board (SERB) – Starting grants, Infosys Young Investigator grant, and Novo Nordisk Foundation grant, Rajiv Gandhi University of Health Sciences Extramural Grants, India were awarded to the N.R.G. lab. We were supported by DST-FIST grants and Longevity India Initiative. A.M. was supported by a fellowship from DBT and Prime Minister’s Research Fellowship (PMRF) for pursuing her PhD.

## Author contributions

Conceptualization, N.R.G.; methodology, A.M., V.R., I.B., A.D., and N.R.G.; software, A.M.; validation, A.M., V.R., I.B., and A.D.; formal analysis, A.M., V.R., and I.B.; investigation, A.M. and N.R.G.; resources, A.M. and N.R.G.; data curation, A.M.; writing – original draft, A.M., V.R., I.B., A.T., and N.R.G.; writing – review and editing, A.M., V.R., I.B., A.T., A.D., and N.R.G.; visualization, N.R.G.; supervision, N.R.G.; project administration, N.R.G.; funding acquisition, N.R.G.

## Declaration of interests

The authors declare no competing interests.
