# Supplementary material for: Protocol for the detection of large dense-core vesicle exocytosis using an automated image-processing algorithm
Source: STAR Protoc. 2025 Dec 10;6(4):104264. doi: 10.1016/j.xpro.2025.104264 (PMC12756630; doi:10.1016/j.xpro.2025.104264)
Supplement: Document S1. Figure S1 [file mmc1.pdf]

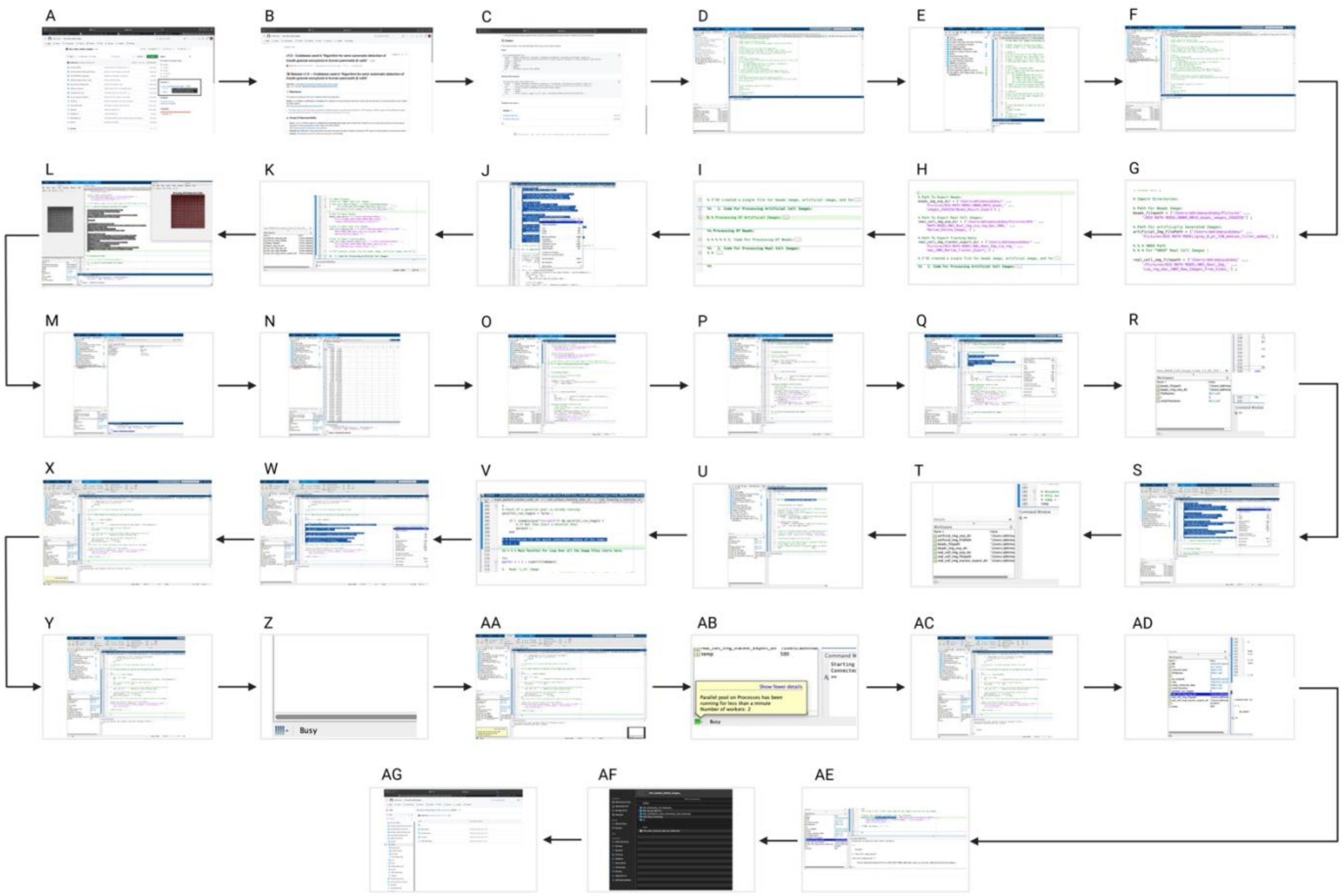

Supplementary figure 1: A step-by-step guide for the usage of the algorithm.

1. **Code Installation:**
   Download version v1.0 from the github repository with the following link:
   a. https://github.com/dabhimanyu/bio_math_model_images.git
   b. Screenshots depicting the steps is given in supplementary figure 1.
   c. Click on v1.0 in the release section.
   d. 0002 screenshot will appear.
   e. Go to the bottom in the assets section. (Sup. fig. 1C)
   f. Download the zip file
   g. Extract its contents in a folder and open this folder in MATLAB.
   h. In the command space type " addpath(genpath(pwd)) " and hit enter (Sup. fig. 1D)
   i. Notice the folders on the left are now within the path.
   j. You are now all set to run the code. (Sup. fig. 1D and E)

2. How to run the code:

a. Open the mFile "new_MAIN_Cell_Image_Code_13_06_2022_" (Sup. fig. 1E)

b. Feed in all the import directories. You don't need to feed in all of them. Just the ones you want to work on. (Sup. fig. 1G)

c. Ditto for export directories (Sup. fig. 1H)

3. Running Artificial Image Code:

a. Comment out all other parts of the code that you don't want to work on.

b. For ex, here (Sup. fig. 1I,) except for the Artificial Images, the rest two sections would be commented out, as shown in Sup. fig. 1J.

c. Code has been divided into sections by making use of %%. Any section could be run by pressing control enter. You you can simply select the code that you want to run, right click and then select “Evaluate selection in command window”. (Sup. fig. 1J)
d. Here you’ll be selection the first section and then run it. (You are in inspection stage, to see if the code is working or not. So you’ll be running your code partially to see if it’s working. Once everything is done then you’ll run the whole code.
e. So first run the first section of the code to initialise the variable path.
f. Once you do that, you should see the names of the variable which declares your input and output directories (Sup. fig. 1K)
g. Once the path is initialised, you come to the section of code that you want to run. Here it’s the Code for the artificial Images.
h. Select the section of code for the artificial images and run it in the same fashion as previously. Or since the code has already been sectioned so you can simply press control enter to run this part of the code. (Sup. fig. 1L)
i. YOu’ll see the results. (Sup. fig. 1L) (run “figure ; imshow(im_original)” to also see the original image to compare them side by side)
j. Particle Centres or centroids and the number of particles data are stored in variable CC. (Sup. fig. 1M and N)
k. Similarly the code for the beads image could be used.

4. Running Cells Code:
a. Like before, run the first section of the code to set up the file path. Either select the code, right-click click and then “Evaluate selection in command window”.
b. Select and run the section corresponding to the Code For Processing Real Cell Images:(Sup. fig. 1T and U)
c. The “parallel_run_toggle” is off by default. Turn it on if you want to do a parallel run.
d. By default, i = 1 is selected (Sup. fig. 1V). Before running the TIRF code on the full image stack, test it on one image.
e. Select the code within the loop, right-click and run it in the command window. (Sup. fig. 1W). This gives you a check if everything is working fine or not.
f. Once everything checks out then you can run the complete loop. It’ll figure out if you have Parallel Computing Toolbox (PCT) or not. If you have it’ll run in parallel mode if not it’ll automatically run in the serial mode. It won’t throw any error. Sup. fig. 1X – 1AC.
g. The output shows all images (500 images in this case) were processed in less than a minute. (Sup. fig. 1AC)
h. Centroid data for all of the frames is stored in ‘real_cell_img_exp_dir’ the path you had initially given. (Sup. fig. 1AD)
i. Centroid data could be found in the export directory (Sup. fig. 1AE and AF)
